# Unlocking the therapeutic potential of primary tumor-draining lymph nodes

**DOI:** 10.1007/s00262-019-02330-y

**Published:** 2019-04-03

**Authors:** Jossie Rotman, Bas D. Koster, Ekaterina S. Jordanova, A. Marijne Heeren, Tanja D. de Gruijl

**Affiliations:** 1 Department of Obstetrics and Gynecology, Center for Gynecological Oncology Amsterdam (CGOA), Amsterdam UMC, Cancer Center Amsterdam, Vrije Universiteit Amsterdam, Amsterdam, The Netherlands; 2 Department of Medical Oncology, Amsterdam UMC, Cancer Center Amsterdam, Vrije Universiteit Amsterdam, De Boelelaan 1117, 1081 HV Amsterdam, The Netherlands

**Keywords:** Tumor-draining lymph node, Local immunotherapy, Cervical cancer, Melanoma, Breast cancer, TIMO 2018

## Abstract

Lymph nodes draining the primary tumor are essential for the initiation of an effective anti-tumor T-cell immune response. However, cancer-derived immune suppressive factors render the tumor-draining lymph nodes (TDLN) immune compromised, enabling tumors to invade and metastasize. Unraveling the different mechanisms underlying this immune escape will inform therapeutic intervention strategies to halt tumor spread in early clinical stages. Here, we review our findings from translational studies in melanoma, breast, and cervical cancer and discuss clinical opportunities for local immune modulation of TDLN in each of these indications.

## Introduction

Many complex processes are involved in the metastatic spread of cancer cells from the primary tumor to lymph nodes and distant organs. The sentinel lymph node (SLN) is the first node to receive lymphatic drainage from the primary lesion and is of great importance in initiating an effective anti-tumor immune response; it also constitutes a first line of defense against metastatic spread [1]. For many malignancies, the presence of tumor cells in tumor-draining lymph nodes (TDLN), and in the SLN in particular, is a key prognostic factor and, in some cases, predicates the course of treatment [2]. In some tumors, e.g., cervical cancer (CxCa) or oral cancer, a complete lymphadenectomy provides overall survival benefit [3–6]. However, for other indications, such as melanoma [7] and breast cancer (BrC), this is not the case [8].

### Tumor-draining lymph nodes as a target for immunotherapy

The main focus of current immunotherapeutic strategies is on targeting the microenvironment of primary tumors and/or metastatic lesions, most notably by checkpoint inhibitors. As therapeutic targets, TDLN, and SLN in particular, are relatively undervalued, and clinically under-utilized. They are, nonetheless, essential players in anti-tumor immunity. In this focused review, we will discuss the importance of, and clinical opportunities for, therapeutic targeting of TDLN, based on findings from pre-clinical and clinical studies carried out by our group.

In the TDLN, tumor-specific T-cell responses are initiated. Here, effective priming of cytotoxic CD8^+^ T cells takes place upon tumor-specific (neo)antigen recognition, presented by APC, including DC and macrophages [9]. Although DC represent only a small population of all the immune cell subsets in the LN, they are crucial in initiating an effective immune response. In cancer, however, TDLN are under the influence of tumor-derived factors, such as extracellular vesicles [1], IL-6 [10], TGF-β [11], prostaglandin-E2 (PGE2) [10], and VEGF [12, 13]. As a result, DC are suppressed and acquire an immature and M2 macrophage-like phenotype, and will, therefore, not properly cross-present in TDLN [14]. During tumor progression and prior to metastasis, TDLN undergo many additional profound alterations leading to invasion by cells derived from the primary tumor [1, 2, 15]. Such alterations include increased lymphangiogenesis, blood vessel remodeling, and increased chemokine and cytokine secretion, which can ultimately lead to changes in immune cell composition, resulting in a ‘tumor-supportive’ microenvironment, i.e., the pre-metastatic niche [1]. Moreover, with the ability of tumor cells to evade immune surveillance by the upregulation of immunosuppressive ligands and downregulation of MHC class I-molecules, this can eventually lead to the metastatic growth of tumor cells that have reached the TDLN [1].

Thus, immune modulation of TDLN could generate effective tumor-specific T-cell responses and in this way prevent metastatic spread. Considering that only a minor fraction of systemically administered drugs reaches the TDLN [16], locally applied therapies may be more effective in counteracting immune suppression in TDLN. Based on immune profiling and ex vivo proof-of-concept studies, we have conducted and are currently conducting a number of clinical trials aimed at immune potentiation of the TDLN through local delivery of immune modulatory drugs.

### Immune profiling of lymph nodes in cancer

Over the past 2 decades, our group has pioneered the flow cytometry-based immune profiling of TDLN in humans. In these studies, we employ a scraping method (i.e., we scrape the cutting surface of a bisected TDLN) to obtain viable leukocytes from the TDLN, which was shown not to interfere with diagnostic procedures [17]. Compared to dissociation of the entire node, we found similar viabilities and phenotypic characteristics of T-cell and DC subsets in scrapes [18]. In addition, using multiparameter (fluorescent) IHC, we are currently working on improving our understanding of the TDLN architecture and cellular networks by studying (co-)localization of diverse immune cell subsets in their microenvironment [19–21].

### The influence of primary and invasive melanoma on conventional DC in SLN

In early pioneering studies on the immune status of melanoma SLN, Cochran and colleagues convincingly demonstrated that DC in SLN were more immune suppressed than DC in further downstream located TDLN [22, 23]. This observation suggested DC to be a prime target of melanoma-induced immune suppression, consistent with their pivotal role in initiating T-cell-mediated anti-tumor immunity. Our group was the first to characterize and functionally test conventional DC (cDC) subsets, distinguishing migratory from LN-resident (LNDC) subsets, in human skin-draining LN using multiparameter flow cytometry [24] closely followed by Segura and colleagues [25]. We identified two migratory CD1a^+^ cDC subsets, i.e., dermal DC (DDC) and Langerhans cells (LC), and two LNDC CD1a^−^ cDC subsets, distinguished by absence or presence of CD14 expression (see Table 1). The relative importance and varying roles of these cDC subsets in the priming of immune responses in healthy human LN remains largely elusive, but some clues are emerging. The migratory subsets take up antigens in the skin and will subsequently migrate to the skin-draining LN, where they can present those antigens to T cells. The two LNDC subsets are found in skin-draining LN but not among DC migrated from skin explants and are recruited from the peripheral blood to the LN [26]. They are key players in cross presentation as evidenced by the high surface levels of cross-priming associated markers CLEC9A and BDCA3/CD141 (as well as expression of BATF3 mRNA; van de Ven et al., unpublished data) and by correlation of their frequencies to cross-presentation ability of melanoma SLN single-cell suspensions, which we observed after TLR9-mediated conditioning [26]. Importantly, although the migratory subsets appeared more phenotypically mature under steady-state conditions, ex vivo isolated LNDC (both CD14^−^ and CD14^+^) subsets proved more powerful in vitro primers of allogeneic effector T cells, which might tie in with higher release levels of T-cell-activating cytokines [24]. Functional differentiation between the CD14^−^ and CD14^+^ subsets remains obscure, but both may be involved in the priming of systemic anti-tumor effector T-cell responses, as we found the activation state of either to be associated with distant recurrence-free survival in early stage melanoma [27]. Another DC subset in skin-draining LN are plasmacytoid DC (pDC) [26]. These cells are poor antigen presenters, but are powerful producers of type I interferons upon TLR activation [28]. As such, pDC play an important role in the activation of cDC and other immune cells.

**Table 1:** Conventional dendritic cell subsets found in skin-draining lymph nodes

Name	Phenotype [24]	Origin [24]	Most affected by [27]
Langerhans cells	CD1a^hi^CD11c^int^	Skin (migratory)	Primary tumor
Dermal dendritic cells	CD1a^int^CD11c^hi^	Skin (migratory)	Primary tumor
CD14^−^ LNDC	CD1a^−^CD11c^+^BDCA3^hi^CD14^−^	Circulation (LN resident)	LN metastasis
CD14^+^ LNDC	CD1a^−^CD11c^+^BDCA3^lo^CD14^+^	Circulation (LN resident)	LN metastasis

In melanoma, we observed a significant negative correlation between the activation state (based on CD83 expression) of DDC and LC in the SLN and primary tumor burden (Breslow thickness) [27]. Interestingly, primary tumor burden was not shown to have a significant effect on either the frequency or activation state of LNDC subsets. However, the presence of SLN tumor metastases did have a significant impact on both the frequency and activation state of conventional LNDC, the latter showing a reverse correlation with the size of the metastasis (Table 1). This suggests that the primary melanoma can create a pre-metastatic niche in the TDLN by suppressing the activation states of migratory cDC subsets, which was shown to be associated with a shorter local recurrence-free survival. Subsequently, TDLN metastasis suppress LNDC which, interestingly, was shown to be associated with a worse distant recurrence-free survival [27]. The latter indicates an essential role for conventional LNDC in the induction of effective systemic anti-tumor immunity.

### Immune modulation of the melanoma SLN

The 10-year melanoma-specific survival of stage I and II melanoma patients, defined as any primary tumor without regional or distant metastases, ranges from 98 to 75% depending on risk factors, such as Breslow tumor depth and tumor ulceration. After tumor spread to the regional LN, the 10-year melanoma-specific survival can drop to as low as 24% in patients with stage IIID melanoma [29]. The unmet medical need for many of these patients stems from the fact that there is no widely used adjuvant treatment available to reduce the chances of disease recurrence, although systemic treatment (neo-adjuvant, i.e., preceding complete lymph node dissection) with immune checkpoint inhibitors in patients who are at very high risk of recurrence (high-risk stage III) and treatment with dual BRAF and MEK inhibitors in patients with BRAF V600E or V600K mutated stage III melanoma, has shown to improve recurrence-free survival [30–33], and has recently been approved by the FDA. For all other early stage patients, there is a “wait and see” approach after surgical removal of the primary lesion and SLN.

**Fig. 1:**
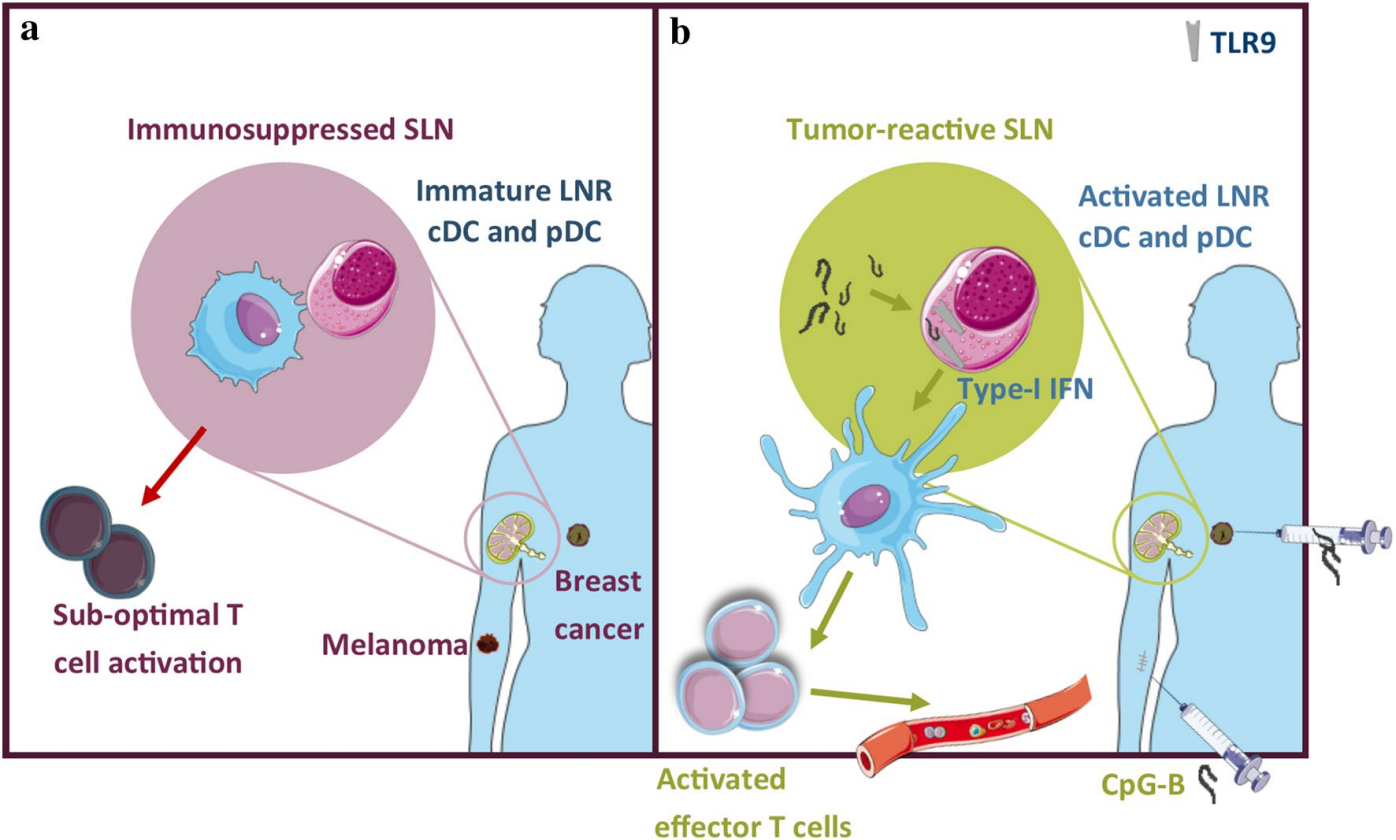
In melanoma and breast cancer draining SLN, tumors can effectively suppress the activation state of LN-resident cDC (**a**). Immune modulation of the SLN by local injection of TLR-9 agonist CpG-B results in activation of LN-resident cDC subsets (through type-1 IFN release by pDC), which ultimately leads to systemic protection against later tumor recurrences (**b**)

Interestingly, we were able to show in multiple randomized and placebo-controlled clinical trials that there is a good rationale to treat these early stage melanoma patients with local immunotherapy aiming to prevent loco-regional and, eventually, distant spread, while minimizing immune-related side effects in this essentially healthy population. Our earliest results were published in 2004 and reported on a 2-armed (1:1) randomized placebo-controlled phase II trial in which 12 patients received four daily intradermal injections directly adjacent to the scar from the primary melanoma excision from day − 3 to day 0, just before the sentinel node biopsy (SNB) and re-excision of the (former) primary tumor site. Patients received either 3 μg/kg body weight recombinant human GM-CSF dissolved in 1 mL saline or 1 mL plain saline alone. GM-CSF administration resulted in higher frequencies and enhanced maturation and activation state of CD1a^+^ migratory cDC in the SLN [34]. In two more recent trials, we showed that low-dose intradermal injections with the TLR9 agonist CpG-B, either alone or combined with GM-CSF, at 1 week prior to the SNB, resulted in enhanced activation of conventional CD14^−^ and CD14^+^ LNDC as well as of pDC in the SLN [26, 35]. Interestingly, this local immunotherapy instigated local (i.e., in the SLN) as well as systemic tumor-specific CD8^+^ T-cell reactivity [36]. A recent meta-analysis showed that patients in the treatment arm of these two studies had fewer tumor-positive SLN after SNB and a longer recurrence-free survival [37]. These studies thus deliver an important proof-of-concept, showing that local immune modulation, specifically of TDLN, may lead to systemic protection against later tumor recurrences (see Fig. 1). We are currently planning a confirmatory randomized and placebo-controlled phase II clinical trial with a next-generation CpG oligodeoxynucleotide in 214 patients with stage II melanoma (Netherlands Trial Registry no. NTR7355).

### Immune modulation of TDLN in breast cancer

Comparable DC-targeting therapeutic approaches may be implemented in patients with BrC, since both melanoma and BrC drain to LN in the skin catchment area with comparable migratory and LN-resident DC subset distribution profiles. In BrC, neoadjuvant chemotherapy (NAC) is one of the treatment options. A pathologic complete response (pCR) upon NAC is an independent predictor for favorable clinical outcome in all molecular subtypes [38]. Interestingly, T-cell infiltration in BrC holds predictive value for response to chemotherapy [39]. Since certain cytostatic drugs can induce immunogenic cell death (ICD), leading to the release of tumor-associated antigens [40], there is a clear rationale to combine NAC with DC-potentiating strategies to optimize tumor-specific T-cell priming in the TDLN. An early study from 1999 already showed a favorable effect on patient survival of combined GM-CSF with NAC in patients with locally advanced BrC [41]. Patients were treated with doxorubicin, cyclophosphamide (both agents known to induce ICD) and GM-CSF at three-weekly intervals. After a maximum of six cycles, patients underwent surgery and postoperative radiotherapy. We observed higher frequencies of mature DCs in the TDLN of these patients, suggesting that GM-CSF is able to improve patient outcome via DC recruitment and maturation, and a subsequent anti-tumor response [42]. Interestingly, we have observed a similar relationship between hampered activation of LNDC and tumor involvement of SLN in patients with BrC as we previously reported in melanoma (van Pul et al. manuscript submitted). Therefore, in analogy to our clinical findings in melanoma, CpG-based local immune potentiation in combination with NAC may improve response rates in patients with BrC. This certainly deserves further (pre-)clinical exploration.

### The role of TDLN in cervical cancer

In contrast to melanoma and BrC, CxCa is caused by a persistent infection with high-risk strains of the human papillomavirus (HPV), mainly HPV16 and HPV18. HPV-specific T cells [43] as well as T cells that target non-viral tumor-associated (neo-)antigens [44] have been detected in CxCa TDLN. As HPV-derived antigens are highly immunogenic, it is assumed that an immunosuppressive environment facilitates immune escape and thereby causes lymphatic spread.

CxCa is a locally invading disease and initially metastasizes to pelvic TDLN. The presence of LN metastases in patients with CxCa is a crucial prognostic factor [45]. Importantly, survival benefit was observed for CxCa patients who underwent complete lymphadenectomy upon low-volume disease detection in the SLN, or even upon the removal of solely tumor-negative LN [3, 5], indicating the presence of an unfavorable immune microenvironment in CxCa-draining pelvic LN. To understand the cellular basis for this phenomenon and to find new immunotherapeutic targets that would allow immune stimulatory conversion of the TDLN microenvironment, we performed several studies in which we found various immune escape mechanisms exploited by CxCa.

### The influence of PD-L1^+^ M2-like macrophages on cervical cancer progression

Interestingly, flow cytometric characterization of diverse immune cell subsets in TDLN of CxCa patients, showed that in contrast to melanoma and BrC, Langerhans cells were hardly present in CxCa LN. Although higher levels of CD1a^+^ DCs were present in tumor-positive LN (LN+) as compared to tumor-negative LN (LN−) [46], these cells might have been derived from recruited and tumor-converted monocytes rather than conventional migratory CD1a^+^ DC. Remarkably, we did not find evidence of decreased LNDC activation. These results point to the requirement for a different immunotherapeutic approach aimed at TDLN conditioning in CxCa, than the one tested and proposed for melanoma and BrC, respectively.

In addition to higher levels of CD1a^+^ DCs, elevated levels of activated CD8^+^ T cells in LN+ suggested immune activation [46]. However, this activation was apparently overruled by a highly immunosuppressed microenvironment in LN+ compared to LN−, with high expression levels of the checkpoint molecules PD-1 and CTLA-4 on T cells and the presence of MDSC. Moreover, high rates of Tregs were observed in LN+, which correlated with the rates of M2-like CD14^+^PD-L1^+^ APC. A cytokine release profile consistent with an immune suppressive microenvironment was observed as well, with high IL-10, IL-6, TNFα, and low IFNγ expression. In a comparative study of all dissected cervical TDLN from five patients with CxCa, we found that immune suppression (identified as low CD8^+^ T cell/FoxP3^+^ Treg ratios) preceded actual metastasis, creating metastatic niches in the tumor-draining lymphatic catchment area [21]. We hypothesize that primary tumors are able to recruit (possibly via the secretion of CCL2) [47] and polarize CD14^+^ monocytes into suppressive PD-L1^+^ M2-like macrophages [(co)-expressing CD14 and/or CD163] [48]. These M2-macrophage-like cells, induced by tumor-derived factors, are incapable of stimulating proper CD8^+^ T-cell responses, favor Treg expansion, and facilitate tumor progression by the production of pro-angiogenic and pro-tumor-invasive factors [14, 49].

In aggregate, our findings support the clinical exploration of immunotherapies in CxCa aimed at converting the prevailing immunosuppressive conditions in the primary tumor and TDLN into an immune-activated tumor-targeting environment.

### Modulating TDLN in cervical cancer

**Fig. 2:**
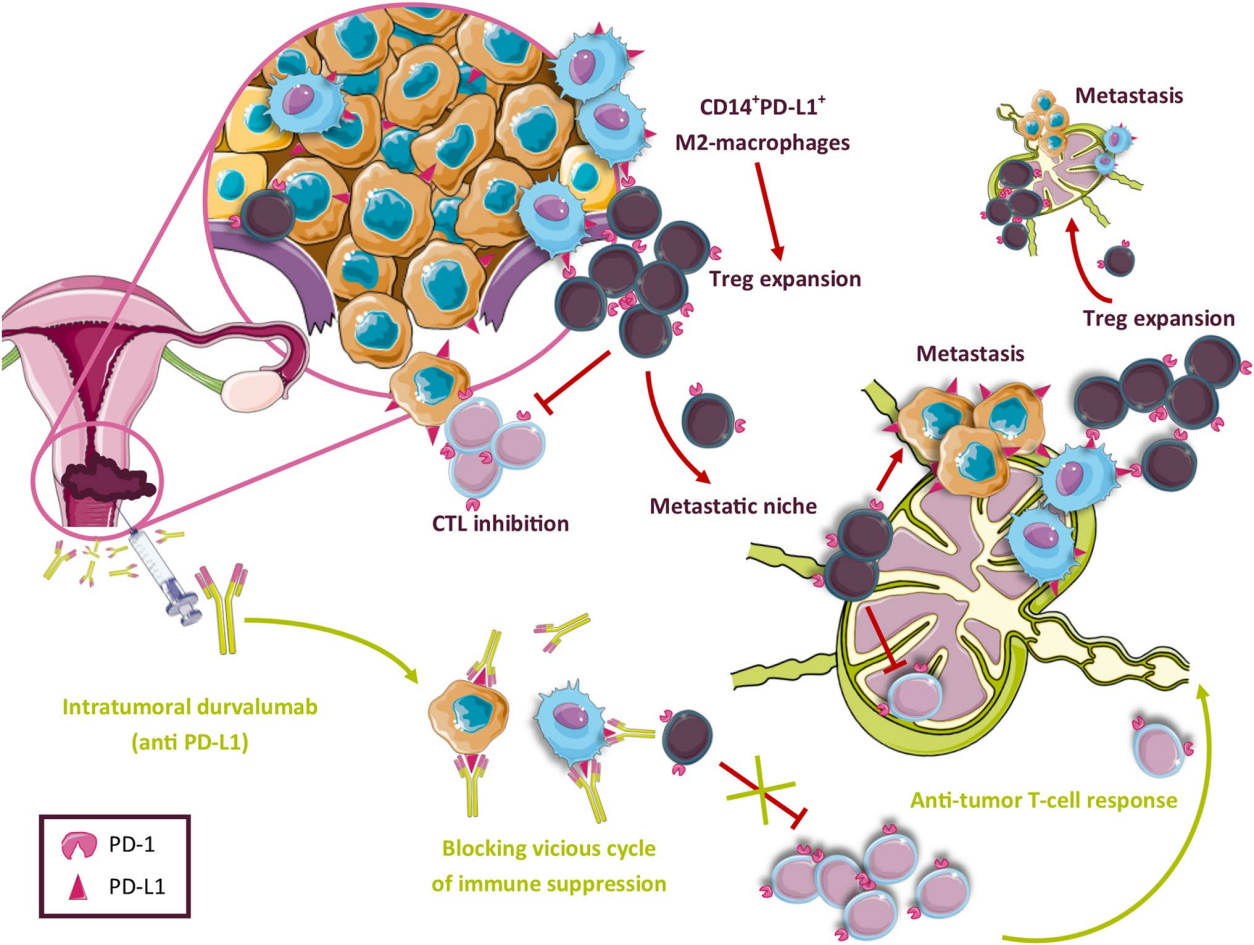
Model of tumor-related immune suppression in CxCa TDLN. In the primary tumor expansion and activation of Tregs takes place through their interaction with CD14^+^PD-L1^+^ M2-like macrophages (differentiated from monocytes recruited to the TME from peripheral blood). This leads to effector T-cell suppression and, upon their migration, to a pre-metastatic niche formation by Tregs in the first-line CxCa TDLN. Upon subsequent metastatic spread, monocytes are again recruited to the TME and converted into immunosuppressive M2-macrophages. These in turn expand and activate a new wave of Tregs that migrate to more distant TDLN and promote further metastatic spread through the LN catchment area. This vicious cycle of immune suppression may be interrupted by blocking the negative impact of PD-L1^+^ M2-like macrophages with intratumorally applied PD-L1 checkpoint blockade

Recently, an immune checkpoint inhibitor of PD-1, pembrolizumab, was approved by the FDA for patients with recurrent or advanced CxCa based on an overall response rate of 14.3% and a complete response rate of (only) 2.6% [50]. Based on these results, and the fact that CxCa is mainly a locally invasive disease, we believe that intratumorally administered immunotherapies in earlier stages of CxCa may accomplish tumor control, as TDLN and the PD-L1^+^ macrophages residing therein are most efficiently targeted in this manner. We hypothesize that interference in the functionality of M2-like macrophages in the TDLN may hamper Treg expansion and break the vicious cycle of metastatic niche formation and tumor spread through the lymphatic catchment area, and subsequently to more distant sites (see Fig. 2). Currently, a phase-I clinical trial is ongoing, testing the safety and feasibility of a single low dose of intratumorally injected durvalumab (anti-PD-L1) in CxCa patients 2 weeks before radical hysterectomy with pelvic LN dissection (Netherlands Trial Registry no. NTR6119). With this strategy, we aim to achieve modulation of the microenvironment in the primary tumor and the TDLN and so break immune suppression. This will hopefully result in the generation of both local and systemic tumor-specific T-cell reactivity [51], like we previously observed when investigating locally administered CpG-B in melanoma patients, with an even shorter time window of 1 week between drug administration and surgery.

## Conclusion

Immune profiling of TDLN in patients with various types of solid tumors enabled us to ascertain the suppressive effects of the tumor on loco-regional cellular immunity and provided a clear rationale for the local application of immune modulating therapies targeting TDLN (see Table 2). It is essential to perform immune profiling for each tumor type and subsequently select the appropriate immune modulating agent, as various possible mechanisms of immune suppression were found per tumor type. Importantly, we found evidence of systemic anti-tumor immune activation which seemed capable of preventing (distant) recurrences, as shown by a profoundly increased 10-year recurrence-free survival rate in melanoma patients treated locally with CpG-B prior to the standard-of-care SLN procedure. With the use of less invasive locally applied therapies, surgical complications resulting from LN dissection (e.g., lymphedema) may be avoided. Moreover, this localized therapeutic approach may stop cancer spread in its tracks at an early stage and trigger a protective systemic anti-tumor T-cell response without the unwanted, and sometimes severe, side effects associated with systemic treatment with immune checkpoint blockade [52, 53]. This may have a major impact on patient survival and quality of life. Moreover, by administering a single low dose, the high costs associated with systemic immunotherapeutic treatments in more advanced stages of cancer could be conceivably reduced [54].

**Table 2:** Theoretical advantages of low-dose, local immune potentiation in early stage cancer

1. Low(er) tumor load
2. Low(er) levels of immune suppression
3. Limited tumor heterogeneity: clonal neoantigens [58]
4. Systemic protection against distant recurrence [36, 37]
5. Single administration provides long-lasting protection [37]
6. Limited to no side effects [34, 35]
7. Pre-empts the need for expensive systemic therapies
8. Off-the-shelf generally applicable
9. Leveraging a (sub-optimally) primed T-cell repertoire in the TDLN [36]

In conclusion, we believe that TDLN are of major importance in initiating a robust anti-tumor response upon immune modulating therapies and should be targeted by local delivery of immune modulatory agents. Evidence for this was provided by i.t. delivery of CTLA-4 blocking antibodies in a mouse model, showing equivalent tumor control to systemic administration with reduced side effects [55]. Interestingly, Chamoto and colleagues observed absent anti-tumor efficacy of PD-1 blockade in a mouse model with TDLN ablation, and so demonstrated TDLN to be indispensable, even for an immune modulatory agent assumed to be primarily active in the tumor microenvironment [56]. Fransen et al. recently confirmed these results and, importantly, showed equal in vivo anti-tumor efficacy of low dose locally injected anti-PD-1 and of systemically administered high-dose anti-PD-1 [57].

The rational design of future clinical trials targeting TDLN should encompass combinatorial use of immunotherapeutic agents, such as oncolytic viruses and/or immune checkpoint blocking antibodies. Moreover, it will likely not be limited to the cancer types discussed in this focused review, but may also be applied to other solid tumors proven amenable to immunotherapy, such as, e.g., lung cancer and head-and-neck cancer.

## Abbreviations

BrC: Breast cancer
cDC: Conventional dendritic cell(s)
CxCa: Cervical cancer
ICD: Immunogenic cell death
LN−: Tumor-negative lymph node
LN+: Tumor-positive lymph node
LNDC: Lymph node resident dendritic cell(s)
NAC: Neo-adjuvant chemotherapy
pCR: Pathologic complete response
pDC: Plasmacytoid dendritic cell(s)
PGE2: Prostaglandin-E2
SLN: Sentinel lymph node(s)
SNB: Sentinel node biopsy
TDLN: Tumor-draining lymph node(s)
